# Supplementary material for: Incorporating nonlinearity with generalized functional responses to simulate multiple predator effects
Source: PeerJ. 2022 Aug 18;10:e13920. doi: 10.7717/peerj.13920 (PMC9393008; doi:10.7717/peerj.13920)
Supplement: Supplemental Information 1 — Additional information about how we derived and implemented the size-distributed functional response that is used as an example of how nonlinear prey risk can be incorporated into our generalized functional response framework. [file peerj-10-13920-s001.docx]

Electronic Supplement 1 - Derivation of size-distributed functional response

From: Incorporating nonlinearity with generalized functional responses to simulate multiple predator effects

By: Michael W. McCoy, Elizabeth A. Hamman, Molly A. Albecker, Jeremy Wojdak, James R. Vonesh, Benjamin M. Bolker

When deriving the simple Holling type-II functional response, we assume that a predator spends time equal to (searching time + handling time) = $(1/(aN)+h)$ for each prey it captures and processes, (the searching time is *inversely* proportional to the attack rate $a$ and the prey density $N$). The rate at which prey are killed is the reciprocal of the total time per prey, or $1/(1/(aN)+h)=aN/(1+ahN)$. If there are multiple prey types (we’ll index them by $s$ to suggest size classes), each with attack rate $a(s)$ and handling time $h(s)$, then the overall attack rate (i.e. the rate at which a searching predator encounters a prey item of *any* type) is $A=\sum_{\{s\}} a(s)N(s)$. (This expression assumes that prey categories are discrete: for a continuous size distribution, we would use $A=\int_{s_{\text{min}}}^{s_{\text{max}}} a(s)N(s) ds$, where $N(s)$ is now the density of the prey size distribution.) Note that $A$ is a **total** attack rate, with units of (encounters/time), unlike $a(s)$ which has units of (encounters/(time × prey density)). Assuming the predators have no prey preferences beyond those encoded in $a(s)$, the proportion of each type taken is is $f(s)=a(s)N/A$; the effective handling time is the propensity-weighted average, $H=f(s)h(s)$. Analogous to the expression for the simple Holling-II functional response, the functional response for the entire prey population - the per-predator rate at which prey of any size are killed - is $1/(1/A+H)$. The functional response (per-predator loss rate) for a particular prey category $s$ is

$$\frac{f(s)}{1/A+H}=\frac{a(s)N(s)}{1+AH}.$$

We can extend this to multiple predators, assuming they act independently, by summing the total losses due to each predator species:

$$\text{predation loss}=\sum_{i} P_{i}\frac{a_{i}(s)N(s)}{1+A_{i}H_{i}}.$$

While prey depletion by one predator affects the density and size distribution of the prey and hence the *future* uptake of other predators, this form of predator-predator interaction is automatically taken into account by integrating the dynamical model over time.
