# Supplementary material for: Incorporating nonlinearity with generalized functional responses to simulate multiple predator effects
Source: PeerJ. 2022 Aug 18;10:e13920. doi: 10.7717/peerj.13920 (PMC9393008; doi:10.7717/peerj.13920)
Supplement: Supplemental Information 2 — We searched web of science on May 8, 2018 using the terms “(”multiple pred“) OR (”risk reduction“ & pred) OR (“risk enhancement” & pred) OR (MPE & pred) & experiment”, and retained results for the previous 20 years, leading to 492 papers. We then screened the studies to find those that were experimental, manipulated presence of at least two predators, and had treatments of control, predator mono, and multiple predators. After screening, we retained 121 studies. [file peerj-10-13920-s002.docx]

Electronic Supplement 2 - Literature Review Summary

From: Incorporating nonlinearity with generalized functional responses to simulate multiple predator effects

By: Michael W. McCoy, Elizabeth A. Hamman, Molly A. Albecker, Jeremy Wojdak, James R. Vonesh, Benjamin M. Bolker

We searched web of science on May 8, 2018 using the terms “(”multiple pred*“) OR (”risk reduction" & pred*) OR (“risk enhancement” & pred*) OR (MPE & pred*) & experiment", and retained results for the previous 20 years, leading to 492 papers. We then screened the studies to find those that were experimental, manipulated the presence of at least 2 predators and had treatments of control, predator mono, and multiple predators. After screening, we retained 121 studies from 119 papers.

## Warning: package 'patchwork' was built under R version 4.0.4


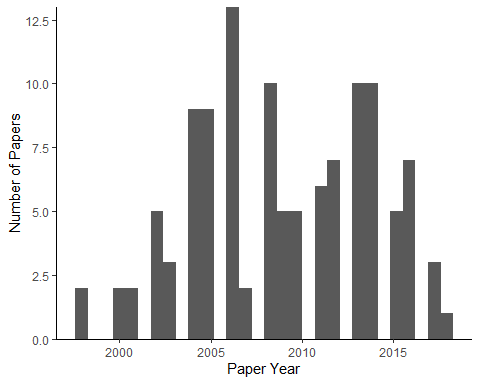


Figure S1: Number of papers included in our summary by year.

# Results

## Do authors report prey size?

75 authors (64%) gave some indication of the size of the prey included in their study, while 45 (36%) did not.


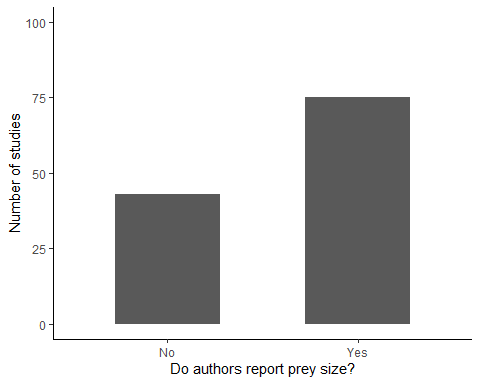


Figure S2: Reports of prey size in multiple predator experiments

## How do authors report prey size?

20 (27%) authors gave a general description of prey size, 30 (40%) referred to a size range, gave some indication of the size of the prey included in their study, while 45 (36%) did not.


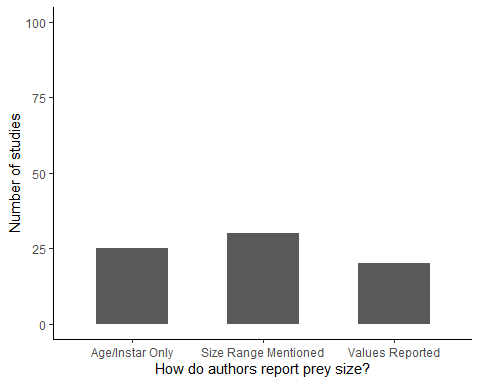


Figure S3: Method of reporting prey size in multiple predator experiments

## Do authors account for prey size in analysis?

Only 24 (20%) of authors accounted for prey in their analysis, typically by analyzing effects for prey of various size classes.


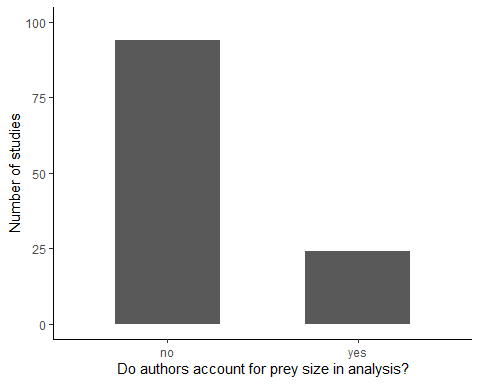


Figure S4: Accounting for prey size in multiple predator experiments
