# Supplementary material for: Incorporating nonlinearity with generalized functional responses to simulate multiple predator effects
Source: PeerJ. 2022 Aug 18;10:e13920. doi: 10.7717/peerj.13920 (PMC9393008; doi:10.7717/peerj.13920)
Supplement: Supplemental Information 4 — Functions and examples demonstrating how to generate multiple predator effects predictions when predation is density dependent, size dependent or both size and density dependent. [file peerj-10-13920-s004.docx]

Electronic Supplement 4 – Code and Repository

From: Incorporating nonlinearity with generalized functional responses to simulate multiple predator effects

By: Michael W. McCoy, Elizabeth A. Hamman, Molly A. Albecker, Jeremy Wojdak, James R. Vonesh, Benjamin M. Bolker

In this supplement we provide functions and examples demonstrating how to generate multiple predator effects predictions when predation is density dependent, size dependent or both size and density dependent. The functions demonstrated here are part of a R library called "Predator.Diversity", which is needed for these functions to work properly. The package is still in development but is available from the authors via GitHub at <https://github.com/eahamman/Predator.Diversity>

1. This model assumes Holling Type II functional responses with size independent attack rates and handling times.

frgrad <- function(t,y,parms) {

with(c(as.list(parms),y),

list(-N0*(a1/(1+a1*h1*N0)+a2/(1+a2*h2*N0)),

NULL))

}

Depletion_model <- function(N0,a1,a2,h1,h2,T) {

L1 <- lsoda(y=c(N0=N0),times=seq(0,T,length=2),

parms=c(a1=a1,a2=a2,h1=h1,h2=h2),

func=frgrad)

(N0-L1[2,-1])/N0

}

Depletion_model(N0=10, a1=0.1, a2= 0.5, h1= 1, h2= 0.9,T= 10)

1. Size-dependent predation with no depletion, assuming size-independent handling time and a unimodal function for attack rate.

parmat <- matrix(c(c1=0.1 ,d1 = 5 ,gamma1 = 0.5 , h1 = 0, , h1 = 0, c2 =0.5, d2 = 10, gamma2 = 2, h2 = 0, h2 = 0), byrow=TRUE, nrow=2, dimnames=list(c("pred1","pred2"),c("c","d","g","m","n")))

svec <- seq(0.1,maxsize,length=50)

timeint=seq(0,1,.1)

Pred_Sims(maxsize=max(svec), dd1=0.001, r1=0.9, prey_dens=100, new_prey=0, pred_dens=c(1,1), predpar.mat=parmat, timevec=timeint, num_steps=10, afun=powRicker, hfun=indh, svec0=svec)

1. Size-dependent predation with depletion, assuming size-independent handling time and a unimodal function for attack rate.

parmat <- matrix(c(c1=0.1 ,d1 = 5 ,gamma1 = 0.5 , h1 = 1, , h1 = 1, c2 =0.5, d2 = 10, gamma2 = 2, h2 = 0.9, h2=0.9), byrow=TRUE, nrow=2, dimnames=list(c("pred1","pred2"),c("c","d","g","m","n")))

svec <- seq(0.1,maxsize,length=50)

timeint=seq(0,1,.1)

Pred_Sims(maxsize=max(svec), dd1=0.001, r1=0.9, prey_dens=100, new_prey=0, pred_dens=c(1,1), predpar.mat=parmat, timevec=timeint, num_steps=10, afun=powRicker, hfun=indh, svec0=svec)

Simulate predation by multiple predators with size-dependent functional responses

**Description**

Simulate predation by multiple predators with size-dependent functional responses

**Usage**

Pred_Sims(maxsize, dd1, r1, prey_dens, new_prey =0, svec0, pred_dens,

predpar.mat, timevec, num_steps, afun = inda, hfun = indh, ...)

**Arguments**

| maxsize | maximum size of prey (all bins will approach this) | |
| --- | --- | --- |
| dd1 | diffusion rate | |
| r1 | growth rate of prey assuming Gompertz growth function | |
| prey_dens | density of prey (one number) | |
| new_prey | number of new juveniles (default is zero, assuming prey are not reproducing during experiment) | |
| svec0 | initial size vector ( a vector of initial sizes with length = number of size classes) | |
| pred_dens | vector indicating the density of each predator species | |
| predpar.mat | matrix of predator functional response parameters (each line is a predator, each column is a parameter) | |
| timevec | vector of timesteps for ode (time between reproduction events) | |
| num_steps | number of overall timesteps (or reproduction events) | |
| afun=inda | attack rate functional form (default is inda=size-independent attack rate). Additional options for size-dependence include: lin (linear), expona (exponential), powRicker (unimodal power-Ricker), hyperbol (Hyperbolic), and modlog (modified logistic) | |
| hfun=indh | handling time functional form (default is indh = size-independent handling time). Additional options for size-dependence include: exponh (exponential), PowFun1 (power function). | |
| powRicker {Predator.Diversity} | |  |

Power Ricker Attack Rate

**Description**

Power Ricker Attack Rate

**Usage**

powRicker(s, c, d, g)

**Arguments**

| s | size vector of prey |
| --- | --- |
| c | maximum attack rate |
| d | size at maximum attack rate (scaling factor along size classes) |
| g | scaler parameter |
